# Isolation of a human-like antibody fragment (scFv) that neutralizes ricin biological activity

**DOI:** 10.1186/1472-6750-9-60

**Published:** 2009-06-30

**Authors:** Thibaut Pelat, Michael Hust, Martha Hale, Marie-Paule Lefranc, Stefan Dübel, Philippe Thullier

**Affiliations:** 1 Groupe de biotechnologie des anticorps, Département de biologie des agents transmissibles, Centre de Recherche du Service de Santé des Armées, La Tronche, France; 2 Institut für Biochemie und Biotechnologie, Technische Universität Braunschweig, Braunschweig, Germany; 3 Integrated Toxicology, United States Army Medical Research Institute of Infectious Diseases, 1425 Porter Street, Frederick MD, USA; 4 IMGT, Laboratoire d'ImmunoGénétique Moléculaire, LIGM, Université Montpellier 2, UPR CNRS 1142, Institut de Génétique Humaine, IGH, Montpellier, France, and Institut Universitaire de France, Paris, France

## Abstract

**Background:**

Ricin is a lethal toxin that inhibits protein synthesis. It is easily extracted from a ubiquitously grown plant, *Ricinus communis*, and thus readily available for use as a bioweapon (BW). Anti-ricin antibodies provide the only known therapeutic against ricin intoxication.

**Results:**

In this study, after immunizing a non-human primate (*Macaca fascicularis*) with the ricin chain A (RTA), a phage-displayed immune library was built (2 × 10^8 ^clones), that included the λ light chain fragment. The library was screened against ricin, and specific binders were sequenced and further analyzed. The best clone, 43RCA, was isolated using a new, stringent neutralization test. 43RCA had a high, picomolar affinity (41 pM) and neutralized ricin efficiently (IC_50 _= 23 ± 3 ng/ml, corresponding to a [scFv]/[ricin] molar ratio of 4). The neutralization capacity of 43RCA compared favourably with that of polyclonal anti-deglycosylated A chain (anti-dgRCA) IgGs, obtained from hyperimmune mouse serum, which were more efficient than any monoclonal at our disposal. The 43RCA sequence is very similar to that for human IgG germline genes, with 162 of 180 identical amino acids for the VH and VL (90% sequence identity).

**Conclusion:**

Results of the characterization studies, and the high degree of identity with human germline genes, altogether make this anti-ricin scFv, or an IgG derived from it, a likely candidate for use in humans to minimize effects caused by ricin intoxication.

## Background

Ricin, a 60 to 65 kDa glycoprotein derived from beans of the castor plant (*Ricinus communis*), is a lectin and member of the A-B toxin family. The B-chain carries the lectin function and binds to specific sugar residues of the target cell surface, allowing ricin to be internalized by endocytosis [1]. The A-chain (RCA-A) has RNA N-glycosidase activity, removing a highly conserved adenine residue in the sarcin/ricin loop of 28S rRNA. The RNA depurination in the ribosome inhibits docking of elongation factor 2, and prevents attachment of amino acids to the polypeptide chain. The result is irreversible inhibition of protein synthesis and eventual cell death [2]. Ricin is on the second priority list of the CDC and is regarded as a high risk for being utilised as a bioweapon.

Ricin is hydrosoluble and, with an estimated LD50 by ingestion of 1 mg/kg in humans, may potentially be used to contaminate food or beverages (reviewed in [3]). Oral intoxications with ricin are encountered in medicine, as accidents usually involving small children, and in suicide attempts among adults. Eight, well-chewed castor beans may be fatal to a 70 kg adult [4]. Ricin administered parenterally to mice has a LD50 of 5 – 10 μg/kg body weight [5]. The toxin has also been administered parenterally to humans, with the most famous case being that of Georgi Markov, a Bulgarian dissident, allegedly killed with ricin in London [6]. Ricin has also been involved in an assassination attempt in Paris [7]. Although oral and parenteral intoxication have been problematic, the intoxication route most feared and the one that causes the most harm, is the pulmonary route. Pulmonary intoxication of ricin shares the same LD50 as the parenteral route [8], but an aerosol delivery can disperse ricin over a larger population and result in damage to many more individuals than stabs in the arm or leg.

Ricin can be produced by individuals using basic equipment and a rudimentary knowledge of chemistry [9]. Since *Ricinus communis *has a world-wide distribution and many plants are grown for decorative purposes, collecting castor bean seeds provides a ready source for toxin production. *R. communis *is also cultivated in the fields to extract, on an industrial scale, castor oil that is used in the chemical industry. By weight, the mash, a side product of oil extraction, can be 1 to 5% ricin. During World War II, large scale production of ricin resulted in the "W" bomb [10]. During the early 1980s, weaponization of ricin occurred in certain states despite being forbidden by international treaties [11].

Until recently, diagnostic and therapeutic tools for identifying ricin intoxication via the lung were not available [3]. This need led our team to develop the first para-clinical test for the diagnosis of pulmonary intoxication caused by ricin [12]. Since then, other investigators have isolated single-domain antibodies in hopes of developing automated detection systems useful for ricin diagnostics [13].

Regarding therapeutics, sophisticated approaches tested thus far have failed to produce synthetic molecules effective for treatment of pulmonary ricin intoxication [14,15]. Additionally, following efforts to produce vaccines inducing neutralizing antibodies against this toxin, two vaccines are under trials but such vaccines may only partially reduce lung damage caused by the aerosolised form of ricin [16]. Previous studies using anti-ricin antibodies of animal origin have, however, shown that these IgGs, in particular when aerosolised, are efficacious in animal models of ricin pulmonary intoxication [17-19]. Thus, at the present time, anti-ricin antibodies provide the primary therapeutic available for ricin intoxication.

Recombinant anti-ricin antibodies should prove useful for both prophylactics and therapeutics in humans. So far however, development of antibodies for human use have only yielded, in a Chinese laboratory, one chimeric antibody thus composed of murine variable regions and human constant regions [20]. This antibody, belonging to a first generation of recombinant antibodies, would very likely induce adverse side effects (HACA, i.e. Human Anti Chimeric Antibodies) and have poor tolerance *in vivo*, due to its murine component. In the present study we isolated, from a non-human primate (NHP) immune library, a human-like scFv, 43RCA, that neutralized ricin very effectively and had a very high affinity for RCA. This affinity is amongst the highest reached for scFvs isolated from any library, without *in vitro *affinity enhancement. In particular, the antibody affinity is approximately 100 times better than previous antibody fragments obtained with the same technique [21-23]. The NHP antibody fragments, like 43RCA, are very similar to their human counterparts, and suitable for "germline humanizing" (or "super-humanizing") [24] in order to ensure their excellent tolerance.

## Results

### Animal immunization

One male macaque was immunised with the A chain of ricin (RCA-A). After five RCA-A injections, the ELISA titre towards whole ricin as an antigen, was equal to 250 000.

### Library construction and isolation of scFv specific for ricin

The fifth (last) boost was given eight months after the fourth RCA-A injection. Three days before the boost, no PCR products could be obtained from the bone marrow using our primers. Amplification was only possible after the last boost and these products were thus regarded to probably code specifically for RCA-A specific antibodies. More precisely, three days after the fifth boost, amplification was detected with 7 of the 9 pairs of primers utilized for amplifying DNA encoding the VH fragment, and with all (7) primer pairs utilized for VLκ. The most diverse DNA was obtained on days 7 and 10, when all pairs of primers strongly amplified DNA that then declined on the 14^th ^day (6) pairs positive for VH and for VLκ). The VLλ primers were tested later using the cDNA originating from the seventh day and MHVL1-f1, MHVL1-f2, MHVL2-f1, MHVL3-f1, MHVL4-f2 and MHVL9/10 (table 1) allowed DNA amplification. All the PCR products amplified from the cDNA obtained on the 7^th ^day were precloned into pGemT, in order to obtain sub-libraries of 7.10^4^, 4.10^4^, and 4.10^3 ^clones for the DNA coding the VH, the VLκ or VLλ fragments respectively. The scFv libraries were constructed in pHAL14 using two cloning steps, starting independently with the VLκ or VLλ sub-libraries. Sizes of the final scFv libraries were 9.4 × 10^7 ^clones for the κ and 8 × 10^7 ^clones for the λ libraries, respectively containing approximately 75% and 95% full-size inserts as determined by colony PCR. Both antibody gene libraries were packaged with Hyperphage and yielded a good scFv surface presentation determined by SDS-PAGE, western blot and anti-pIII immunostain (data not shown). Both libraries were pooled for panning.

**Table 1:** Primers utilized for the amplification of DNA coding for VLλ

MHVL1-f1	5' cag tct gtg ctg act cag cca cc 3'
MHVL1-f2	5' cag tct gtg ytg acg cag ccg cc 3'
MHVL2-f1	5' cag tct gcc ctg act cag cct 3'
MHVL3-f1	5' tcc tat gwg ctg acw cag cca cc 3'
MHVL3-f2	5' tct tct gag ctg act cag gac cc 3'
MHVL4-f1	5' ctg cct gtg ctg act cag ccc 3'
MHVL4-f2	5' cag cyt gtg ctg act caa tcr yc 3'
MHVL5-f2	5' cag sct gtg ctg act cag cc 3'
MHVL6-f1	5' aat ttt atg ctg act cag ccc ca 3'
MHVL7/8-f1	5' cag rct gtg gtg acy cag gag cc 3'
MHVL9/10-f1	5' cag scw gkg ctg act cag cca cc 3'

MHLambdaCL	5' tga aca ttc tgt agg ggc cac tg 3'

Primer names indicate whether they hybridize within the variable (V) or constant (C) region of the DNA.

### Isolation of anti-ricin scFvs

While the library was obtained from a macaque immunized with the RCA-A, it was panned with ricin since the whole toxin was our final target molecule. During panning, the number of eluted phages rose from 10^5 ^(first round of panning), to 5 × 10^5 ^(second round) and finally to 5 × 10^6 ^phages (third and last round). This increase typically indicated enrichment in phages interacting specifically with the antigen. A phage-ELISA utilising ricin and RCA-A as antigens was performed to assess reactivity of the selected phages. Before panning, the signals on both antigens were at the background level, and both signals increased to five-fold over background after the first and the second rounds of panning. According to the phage technology, such signal increases correspond to enrichment in high-affinity binders. After a third round, the signal on RCA-A further increased as expected and it was 15-fold higher than the background, and 25-fold higher on ricin, which would be in agreement with the fact that whole ricin had been utilized for panning. The phagemidic DNA was extracted from the library after its third round of panning, and transformed into *E. coli *HB2151 [25]. Fifty clones were isolated and each monoclonal phagemidic DNA was extracted for sequencing. In parallel, these clones were induced for expression.

### Ricin neutralization in cell-based assays, scFv stability

**Figure 1:**
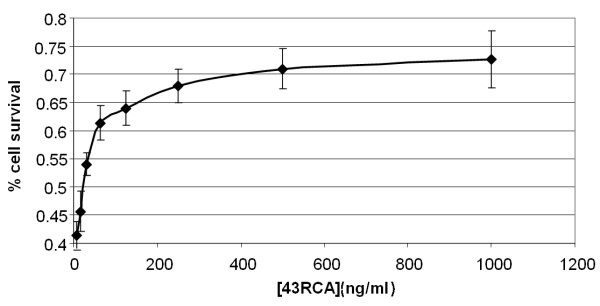
**Ricin neutralization in cell-based assays**. Ricin neutralization capacity of scFv 43RCA, calculated as (signal in average test wells minus signal in 4 no-toxin control wells)/(signal in 4 toxin-only control wells minus signal in 4 no-toxin control wells) and expressed as a function of 43RCA concentration ("ng/ml"). 43RCA IC_50 _= 23 ± 3 ng/ml

Of the 50 clones isolated after the panning, only 19 possessed full-sized, non-redundant sequences that were expressed for further testing *in vitro*. At the highest scFv concentration (1 μg/ml), clone 9RCA showed only 30% inhibition of cellular toxicity even though the molar ratio [scFv 9RCA]/[ricin] was in 150-fold excess. All other clones had neutralizing properties either equal, or less than, clone 9RCA. Exceptions included clones 43RCA, 38RCA, and 44RCA only. By using the cell-based assay, the IC_50 _of clone 44RCA was 484 ± 10 ng/ml and that of clone 38RCA, 93 ± 5 ng/ml. The best clone, 43RCA, had an IC_50 _equal to 23 ± 3 ng/ml (Figure 1), representing a molar ratio [43RCA]/[ricin] equal to 4. This clone was sufficiently expressed (around 100 μg/culture liter) to allow further study.

In order to determine scFv stability, the scFvs were incubated in culture media for 24 h (37°C). The reactivity of clones 43RCA, 44RCA, and 38RCA was not affected by incubation as assessed by ELISA. The stability of these three scFvs generally differed from other scFvs that showed less neutralization capacity, as the latter lost between 0 and 75% of their ability to bind toxin in an ELISA after they were exposed to the same conditions.

### Ricin neutralization in cell-free assays

**Figure 2:**
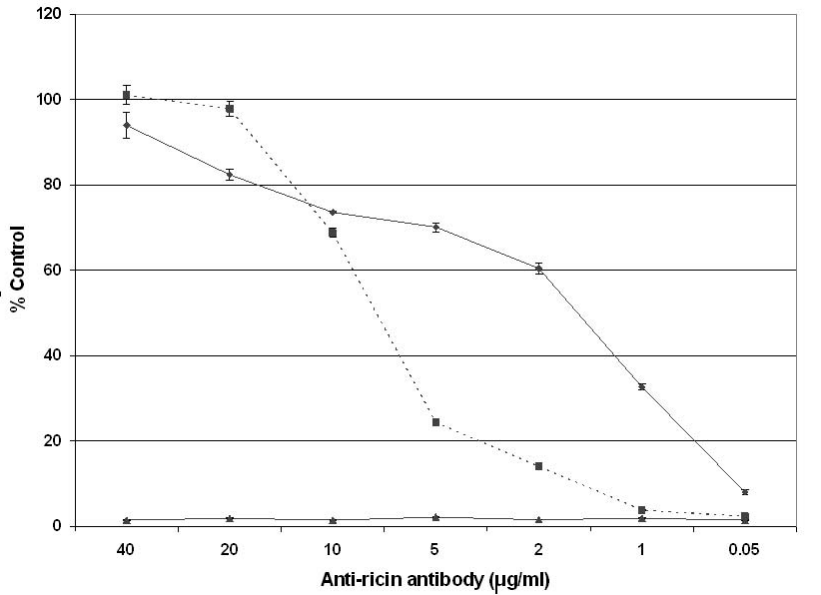
**Ricin neutralization in cell-free assays**. Neutralization of ricin biological activity by 43RCA (diamond, solid line), anti-dgRCA-A IgG (square, dotted line), or non-specific murine Ig (triangle, solid line). Dilutions of the antibodies were mixed with whole ricin and incubated with ricin (4.0 nM) before adding to the translation assay. Luminescence was measured after 90 min at 37°C. Values were calculated as the % control [(CPS treated sample/CPS untreated, no ricin control) × 100]. Results represent the average ± S.D. for three individual experiments.

In order to appreciate whether anti-ricin scFv could function as an effective therapeutic against ricin intoxication, 43RCA was tested for its ability to neutralize ricin biological activity using a cell-free translation assay. The scFv 43RCA neutralized 89% of ricin activity at 40 μg/ml, and even concentrations of 2 μg/ml neutralized 60% of ricin activity when compared to controls containing ricin only (Figure 2). 1.5 μg/ml of 43RCA (50 nM) neutralized 50% of the activity of ricin, present at a 4 nM concentration in this assay, thus the corresponding molar ratio [43RCA]/[ricin] is equal to 12, in the same order of magnitude than for the cell-based assay (4). The activity of 43RCA was compared with the neutralizing capacity of anti-ricin IgGs purified by protein A from sera of mice hyper-immunised with the deglycosylated ricin A chain (anti-dgRCA), the most neutralizing antibodies available to us. By using the same concentrations, the anti-dgRCA IgGs showed ~20% neutralization at 5 μg/ml, and none at 2 μg/ml. The molecular weight of a scFv is 25 kDa with one paratope, while the molecular weight of an IgG is 150 kDa with two paratopes. As a consequence, the molecular weight of a paratope in the form of an IgG is three times larger than that of a scFv, and an IgG concentration of 5 μg/ml represents approximately the same molar paratope concentration as the scFv concentration at 2 μg/ml. At this same concentration of paratopes, the scFv neutralizes ricin three times more efficiently than the polyclonal IgG.

### Affinity determination

**Figure 3:**
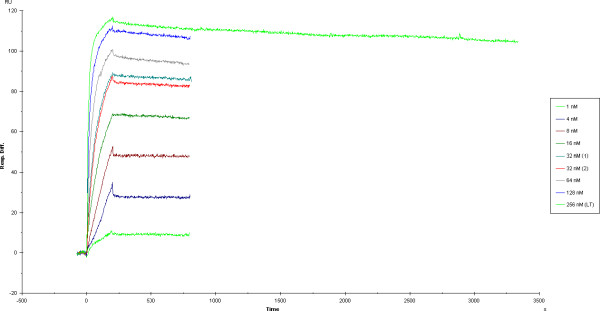
**Sensorgram of 43RCA**. 43RCA affinity was measured at 40.9 pM against ricin, utilizing 4 h elution times; K_on _= 3.34 10^5 ^M ^-1 ^S ^-1 ^and K_off _= 1.36 10^-5 ^s^-1^. The ricin concentrations utilized for the measurement are indicated in the legend box; the solution at 32 nM was tested twice (noted (1) and (2) in the box) and the 256 nM concentration was utilized for the long term elution (noted (LT) in the box).

Affinity measurements focused on the best neutralizing scFvs. The affinity of 43RCA against ricin was initially evaluated in standard conditions, using ~600 seconds elution steps, and resulting in an excellent affinity of approximately 40 pM. The K_D _of 38RCA measured under the same conditions yielded 60 pM. In contrast, the affinity of 44RCA was 4.9 nM, or 100-fold less favourable, although considered high in absolute terms. Because 43RCA neutralisation activity was four times higher than 38RCA, we focused our work upon this more efficient scFv. A more precise measurement of 43RCA's affinity, performed by lengthening the elution time and by using a small scFv immobilization level (100 RU) (Figure 3), gave values of 40.8 pM after a 3 hr elution and 40.9 pM after a 4 hr elution. Measured after a 4 hr elution step, the K_on _was equal to 3.34 10^5 ^M ^-1 ^S ^-1 ^and the K_off_, 1.36 10^-5 ^s^-1^. The quality of these affinity measurements was assessed by the internal consistency test, which had numerical values of 0.56 (3 hr elution measurement) and 0.626 (4 hr elution measurement) when these values have to be inferior to 3 for the measurements to be valid.

### Computational analysis

**Figure 4:**
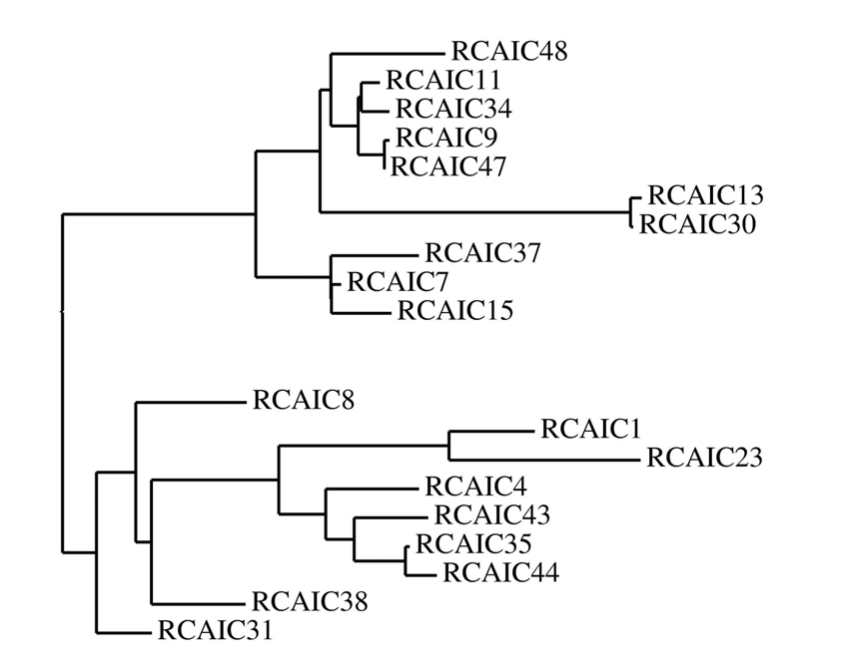
**Grouping of the 19 non redundant scFvs, in the form of phylogenetic tree**. The tree root (extreme left) indicates that the scFvs are grouped in two clusters, A (scFvs 48, 11, 34, 9, 47, 13, 30, 37, 7, 15) on the upper part, and B (scFvs 8, 1, 23, 4, 43, 35, 44, 38, 31) on the lower part of the figure.

Of the 50 isolated clones, only 24 full-sized sequences coding for scFvs could be obtained. On these sequences, 3 were present in 2 occurrences (6 RCA and 37 RCA, 13 RCA and 32 RCA, 14 RCA and 44 RCA) and one was present in 3 occurrences (34 RCA, 41 RCA, 42RCA). Such repetitions may be informative since they could signal scFvs selected due to their high affinity, as was the case with 44RCA. The 19 non-redundant sequences coding the VH and VL domain were automatically analyzed using the IMGT/V-QUEST software. Human germline V, (D) or J alleles, found most similar to these 19 sequences, are shown on tables 2 and 3. In parallel, those sequences were analysed using a new online tool , and this analysis was presented in the form of a phylogenetic tree (Figure 4). The initial root of the tree separated the scFvs in two sub-trees, corresponding to two clusters. Cluster A, corresponding to the sub-tree presented on the upper part of the figure, comprised three subgroups (scFvs 48 RCA, 11 RCA, 34 RCA, 9 RCA, 47 RCA; scFvs 13 RCA, 30 RCA; scFvs 37 RCA, 7 RCA, 15 RCA), whose light chains were of the λ isotype. The 13RCA and 30RCA pair differed only by two nucleotides (A/G41 located in the middle of FR1; and T/C116, the first residues of FR2) and probably resulted from the same parental IgG by somatic hypermutations, which did not lead to a significantly better neutralization effect. Beside their VLλ, 13 RCA and 30 RCA were not related to the rest of cluster A, and were removed from the remainder of the study. Analysis of the [V, (D) and J] genes arranged to code for the 6 remaining scFvs composing cluster A permitted an evaluation of its diversity. Typically, the VH of these scFvs were coded by a unique combination of a single IGHV gene (IGHV4-b) re-arranged with a single IGHD gene (IGHD2-8) and with a single IGHJ gene (IGHJ4) (tables 2 and 3), except for one occurrence in IGHV4-34 (scFv 9RCA) and two occurrences in IGHD1-7 (scFv 37RCA and 48RCA) combined with IGHJ5. The VL of these 6 scFvs were coded by IGLV1-36 and IGLV3-21 on three occurrences each, while another IGLV gene (IGLV1-47) was used twice. The IGLJ genes coding VL were rather constant, with IGLJ1 genes on six occurrences, in addition to one using of IGL3 and IGL6 genes. Overall, the 6 main scFvs in cluster A showed limited diversity and encompassed none of the three best clones. In contrast, cluster B, composed of 8 scFvs (scFvs 1, 23, 4, 43, 35, 44, 38, 31), contained more variability. Six different IGHV genes (1–69, 3–11, 3–23, 3–74, 4–28 and 4–34) were combined with 5 different IGHD genes (3–10, 1–1, 2–8, 6–13, 5–12) and two different IGHJ genes (4 and 5). Similarly, the 8 κ light chains of cluster B were coded by a combination of 7 IGKV genes (1–9, 1–16, 1–17, 1–18, 1–39, 1-NL1, 3–15) and 4 IGKJ genes (1, 2, 3, 4). Thus, detailed analysis of the genes re-arranged to code for the scFvs constituting clusters A and B showed that B had a higher diversity than A. This higher diversity of cluster B was also evidenced by the longer branches of the B subtree as compared to A, and this diversity very possibly allowed cluster B to encompass all the best clones. Such a difference of diversity between clusters is much more easily observed from the tree and subtrees than analysed as detailed above. The presentation of the diversity of antibody sequences in the form of trees could be of further interest when analyzing antibody fragments isolated from a library, even though these trees were not initially designed for this purpose.

**Table 2:** Human germline genes most similar to genes coding for the 10 scFvs belonging to cluster A.

	**VH**			**VL**	
scFv	V	D	J	V	J
RCA7	IGHV4-b*01	IGHD2-8*02	IGHJ4*02	IGLV3-21*01	IGLJ1*01
RCA9	IGHV4-34*13	IGHD2-8*02	IGHJ4*02	IGLV1-36*01	IGLJ1*01
RCA11	IGHV4-b*01	IGHD2-8*02	IGHJ4*02	IGLV1-36*01	IGLJ1*01
RCA15	IGHV4-b*01	IGHD2-8*02	IGHJ4*02	IGLV3-21*02	IGLJ6*01
RCA34	IGHV4-b*01	IGHD2-8*02	IGHJ4*02	IGLV1-47*02	IGLJ1*01
RCA37	IGHV4-b*01	IGHD1-7*01	IGHJ5*02	IGLV3-21*01	IGLJ1*01
RCA47	IGHV4-b*01	IGHD2-8*02	IGHJ4*02	IGLV1-36*01	IGLJ1*01
RCA48	IGHV4-4*01	IGHD1-7*01	IGHJ5*02	IGLV1-47*02	IGLJ3*02
RCA13	IGHV3-11*01	IGHD3-22*01	IGHJ4*02	IGLV1-51*02	IGLJ1*01
RCA30	IGHV3-11*01	IGHD3-22*01	IGHJ4*02	IGLV1-51*02	IGLJ1*01

Human germline genes were retrieved by IMGT/V-QUEST analysis.

**Table 3:** Human germline genes most similar to genes coding for the 9 scFvs belonging to cluster B.

	**VH**			**VL**	
scFv	V	D	J	V	J
RCA1	IGHV1-69*10	IGHD3-10*01	IGHJ4*02	IGKV1-NL1*01	IGKJ1*01
RCA-8	IGHV4-b*01	IGHD1-7*01	IGHJ5*02	IGKV1-18*01	IGKJ2*01
RCA4	IGHV3-23*01	IGHD3-10*01	IGHJ4*02	IGKV1-17*01	IGKJ4*01
RCA23	IGHV1-69*04	IGHD1-1*01	IGHJ4*02	IGKV3-15*01	IGKJ2*03
RCA31	IGHV4-34*13	IGHD2-8*02	IGHJ4*02	IGKV1-39*01	IGKJ2*01
RCA35	IGHV3-23*01	IGHD3-10*02	IGHJ5*02	IGKV1-9*01	IGKJ3*01
RCA38	IGHV4-28*01	IGHD6-13*01	IGHJ5*02	IGKV1-16*01	IGKJ1*01
RCA43	IGHV3-11*01	IGHD5-12*01	IGHJ4*02	IGKV1-17*01	IGKJ4*01
RCA44	IGHV3-74*01	IGHD3-10*02	IGHJ5*02	IGKV1-9*01	IGKJ3*01

Human germline genes were retrieved by IMGT/V-QUEST analysis.

**Figure 5:**
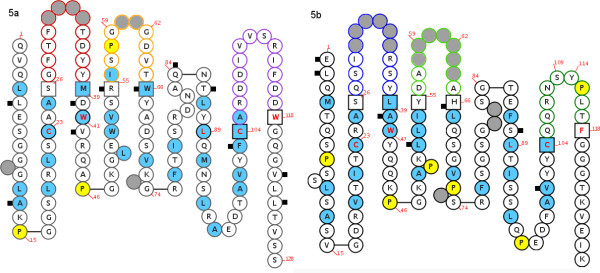
**IMGT Collier de Perles graphical 2D representation of 43RCA (Figure 5a: VH fragment, Figure 5b: light chain)**. IMGT Colliers de Perles representations are displayed according to the IMGT unique numbering. Black squares indicate differences from the human genes most similar to 43RCA, and 43RCA. Positions of hydrophobic amino acids (hydropathy index with positive value, i.e. I, V, L, F, C, M, A) and tryptophan (W) are shown in blue. All proline (P) residues are shown in yellow. The CDR-IMGT sequences are limited by amino acids shown in squares (anchor positions), which belong to the neighbouring FR-IMGT. Hatched circles correspond to missing positions according to the IMGT unique numbering. For the VH domain, CDR1-IMGT is shown in red, CDR2-IMGT in orange and CDR3-IMGT in purple. For the V-KAPPA domain, CDR1-IMGT is shown in blue, CDR2-IMGT in green and CDR3-IMGT in turquoise.

The 43RCA scFv was the most efficient scFv for neutralization of ricin biological activity, and had very similar human germline counterparts as identified by IMGT/V-QUEST software. The overall identity between 43RCA eight framework regions and their most similar peptidic sequences, coded by human germinal genes, was 90%. In the IMGT Collier de Perles representation of 43RCA (Figure 5), black squares indicated amino acids that differ from the closest human germline genes IGHV3-11*01 and IGHJ4*02 for VH and IGKV1-17*01 and IGKJ4*01 for VL. By using IMGT amino acid classes [26], an analysis of the physicochemical properties of these differing 43RCA framework residues was performed and its results summarized in table 4. In particular, the proximity of 43RCA with sequences coded by human germline genes was further appreciated by the fact that only four residues, three located in VH (S40>D and Y55>R in FR2-IMGT, T122>V in FR4-IMGT) (human>macaque) and one in VL (T101 > V in FR3-IMGT) on a total of 180 framework residues, were rated as very dissimilar.

**Table 4:** Localisation and evaluation of differences between 43RCA framework regions, and those coded by 43RCA most similar human germline genes

	Total	Identical	Very similar (+++)	Similar (+-+), (++-), (+-+)	Dissimilar (--+), (-+-), (+- -)	Very dissimilar (- - -)
VH						
FR1-IMGT	25	23	0	2	0	0
FR2-IMGT	17	14	0	1	0	2
FR3-IMGT	38	34	0	1	3	0
FR4-IMGT	11	9	0	1	0	1
**FR-IMGT**	**91**	**80**	**0**	**5**	**3**	**3**
V-KAPPA						
FR1-IMGT	26	24	1	1	0	0
FR2-IMGT	17	15	0	0	2	0
FR3-IMGT	36	33	0	1	1	1
FR4-IMGT	10	10	0	0	0	0
**FR-IMGT**	**89**	**82**	**1**	**2**	**3**	**1**
**All FR-IMGT VH and V-KAPPA**	**180**	**162**	**1**	**7**	**6**	**4**
	**(%)**	**90%**	**0.56%**	**3.89%**	**3.33%**	**2.22%**

Differences between 43RCA VH and VL framework amino acids, and those coded by most similar human genes, are evaluated on a 5-level scale (from identical to very dissimilar, see text) and located in each framework region.

## Discussion

By immunizing with the chain A of ricin (RCA-A), a toxin subunit, we developed a non-human primate (NHP) immune library screened with whole ricin, and we isolated an scFv with picomolar affinity for ricin.

When immunizing with RCA-A, a plant protein that is presumably very different from the macaque self proteins, we anticipated our antigen would be strongly recognised as foreign and elicit a strong immune response. After three injections however, assessment of anti-ricin antibody via an ELISA with RCA-A as the antigen resulted in unexpectedly low titres (in the range of 1:20,000). After a 4th injection, serum was again tested using an ELISA with both RCA-A and whole ricin as antigens. Although RCA-A was the immunogen, titres against RCA-A were 7-fold lower than those against ricin. The differences were probably due to the conformational changes of the subunit when coated onto the plate. The titre measured against whole ricin was 1:250,000 after the fourth injection, indicating a strong antibody response in this NHP. This hypothesis was confirmed when amplification products were obtained with our antibody-specific primers (macaque κ and γ specific) 3 days after the last (fifth) injection, the earliest products we had ever obtained. All pairs of primers allowed amplification 4 days later (7^th ^day post-injection). When compared to our previous work with *Bacillus anthracis *protective antigen (PA) in which the optimal amplification was obtained 35 days after the last immunization, these high responses obtained as early as 3 days after the last immunization were interesting and significant. We hypothesized that this early response, obtained from immunogen-stimulated lymphocytes at the beginning of antigen circulation and thus present at low concentrations, corresponded to the existence of high-affinity surface immunoglobulins that would later be secreted by these lymphocytes. A high library diversity is however necessary to encompass scFvs with the desired specificity (here, directed against neutralizing epitopes). We chose, in order to build the library, the PCR products obtained on the 7^th ^day because, at this still early time after the boost, all PCR primers permitted amplification. For the first time in this study, we also utilized primers designed to amplify VLλ-coding DNAs. The cDNA, that had been previously extracted from bone marrow on the two best days, had been stored (-20°C) and was re-utilized: 5 out of the 11 human λ primers permitted amplification, though more weakly than the macaque κ primers. As a consequence, VLλ pre-cloning was rather inefficient and the size of the VLλ sub-library was ten times smaller than the VLκ library.

After the pre-cloning and cloning steps, the size of the pooled (VLλ and VLκ) anti-ricin library was 2 × 10^8 ^clones; therefore, it was not significantly higher than our former anti-Lethal Factor (LF) library (10^8 ^clones), and this size could not explain the high affinities encountered in this study [21,22]. Because we wanted to search for scFv that would interact with the whole toxin and not A chain alone, and also because of the conformationnal change of RCA-A when coated onto ELISA plates, the library was panned against whole ricin. After panning, while 50 clones were isolated for sequencing, only 19 full-sized, non-redundant sequences were obtained. Approximately half of these sequences had VL of the λ isotype, despite the small size of the VLλ library, probably indicating interest of the λ primers, which were specially designed for the present library construction. These 19 clones were expressed and initially tested for neutralization as the neutralizing activity, not affinity, was a goal of the present study. Since there was not a standardized test for ricin neutralization, we chose to establish a cell-based ricin neutralization assay as an adaption of the assay we previously utilized on numerous occasions to assess the neutralization of anthrax lethal toxin [21,22]. This cell-based assay utilised the mouse macrophage J774A.1 cell line (ATCC-LGC, Molsheim, France), which is also sensitive to ricin [27]. We plated J774A.1 cells with ricin (15 ng/ml) for 24 hours to induce approximately 100% cell death This test is stringent because of its long duration that necessitated scFvs of high affinities to form stable scFv-ricin complexes. We also showed that scFv stability was indeed crucial in this test for effective neutralization of ricin.

By using the cell-based neutralization assay, there was only one clone (43RCA) that neutralized 50% of the ricin toxicity with a scFv/toxin molar ratio equal to four. Neutralization by the second best clone (44RCA) was found to be 4 times less efficient. Precise affinity measurements thus focused on 43RCA. To the best of our knowledge, the obtained value of 41 pM is amongst the best affinities obtained directly from any library panning, without the need for further affinity engineering. This value was not an aberration, since the second best clone also had picomolar affinity. These final results were in accordance to our initial hypothesis, supported by the early PCR product amplification, that the immune response to ricin would be strong. The value of 43RCA for therapeutics was further assessed in a cell-free translation assay, where it was favourably compared to IgG obtained from serum of mice hyperimmunized with the dgRCA-A. This polyclonal has a high neutralization capacity and, in particular, it was more efficient than any monoclonal at our disposal. The fact that 43RCA scFv fragment produced *in vitro *had an efficiency three times higher that of hyperimmune polyclonal IgG suggests that this anti-ricin scFv (or the IgG derived from it) could be a therapeutic of choice against ricin intoxication.

The analysis of scFv sequences, by using the new online tool  to build a phylogenetic tree, grouped the scFvs in two clusters (A and B) with A showing less variability than B. The higher diversity of B certainly explains why it encompassed all three outstanding best clones (scFvs 38RCA, 43 RCA, 44 RCA). The diversity of cluster B was quickly appreciated from the branch lengths of the corresponding phylogenetic sub-tree, as opposed to the sub-tree representing cluster A. In the future, these phylogenetic trees could well be a useful guide for the testing of clones eluted from immune libraries, all the more if more numerous sequences are to be analyzed. Systematic analysis of all clones should probably be reserved to highly variable clusters, represented by long-branched sub-trees, while sampling of only a limited number of scFvs originating from the least variable ("short-branched") clusters might be sufficient.

## Conclusion

NHP immune libraries have formerly allowed us to isolate two neutralizing antibody fragments directed at each subunit (PA and LF) of the anthrax lethal toxin. In this current study, we successfully developed an antibody fragment to neutralize another bioweapon (BW), ricin, and this antibody fragment has now been expressed as a full-size IgG, to be tested *in vivo *in the future and possibly utilised for prophylactic or therapeutic purposes. This approach might be re-utilized as antibodies can efficiently neutralize a vast majority of the numerous BW (in particular the most dangerous ones, such as botulinum toxins, plague, anthrax, smallpox, tularaemia and ricin). Our NHP antibody fragments can be "germline-humanized" ("super-humanized") [24] to ensure their excellent bioavailibility and tolerance. Such predicable pharmacokinetics properties are in opposition to the situation of less predictable synthetic molecules, which fail at an alarmingly high rate (90%) during clinical trials. The end result is loss of significant expenditures and time. This failure rate has not, however, prevented studies in search of synthetic molecules against ricin [14,15]. This is despite a situation where the efficacy of antibodies to neutralize ricin (including its aerosolised form) had been well documented [17-19], but only one chimeric anti-ricin antibody was available [20]. Thus, choosing and financing a more risky path towards therapeutics than the isolation of more effective anti-ricin antibodies, typically focused on human antibodies, tends in particular to indicate that the isolation of such human antibodies is somehow difficult.

A similar situation was encountered regarding antibodies directed against the LF subunit of anthrax lethal toxins, in that very few anti-LF antibodies were obtained despite requirements by anthrax experts, while the isolation of many anti-PA antibodies has been financed. The anthrax situation might be particularly revealing, because anti-PA antibodies were, in their large majority, obtained with techniques that started with lymphocytes of anthrax-vaccinated donors. The anthrax vaccine (AVA) lacks LF, thus explaining the rarity of human LF-primed lymphocytes and finally of anti-LF antibodies. Similarly, the scarcity of ricin-primed human lymphocytes certainly explains the absence of human anti-ricin recombinant antibodies. Humans are not naturally exposed to ricin, and there is not a vaccine approved for human use, at present. Naïve and synthetic libraries of human antibody fragments, and mice engineered with human IgG-coding DNA, have been developed to produce human antibodies without the need for lymphocytes from immunized humans. However, it is interesting to note that these tools were not successful, or not utilized, in the two examples quoted above.

In summary of the present study, we have developed improvements in several aspects of our cost-conscious path towards obtaining neutralizing NHP recombinant antibody fragments for clinical purposes. Due to their proximity with their human counterparts, plus the possibility of "super-humanizing" these antibody fragments [24], such reagents would provide well tolerated therapy against ricin, anthrax, as well as other BW agents. In addition, our methodology developed in the current study should be of interest to researchers beyond the field of BW.

## Methods

### Animal immunization

A cynomolgus macaque (*Macaca fascicularis*) (6 kg) was subcutaneously injected with the non toxic A-chain of ricin (RCA-A) purchased from Sigma, Saint Louis, Missouri. The first injection was RCA-A (100 μg per injection) mixed with complete Freud's adjuvant (Sigma) and then RCA-A mixed with incomplete Freud adjuvant for the remaining injections.

Animal housing was performed in facilities accredited by veterinary services. Animals were provided with NHP feed, water ad libitum, and maintained on a 12-h light cycle. The protocol was approved by the CRSSA ethical committee for animal care and use.

### Construction and screening of the anti-ricin antibody gene library

Bone marrow (5 ml) was sampled several times after the last boost in order to isolate RNA using Tri Reagent (Molecular Research Center Inc., Cincinnati, USA) according to the manufacturer's instructions. RNA was retro-amplified, then primers intended for the amplification of DNA encoding macaque Fd and VLκ were utilised as formerly exposed [22]. Later, a set of human λ primers (table 1) was utilised under the same PCR conditions.

PCR products were first cloned in the pGemT vector (Promega, Madison, Wiscontin), according to the manufacturer's instructions, yielding three antibody gene sub-libraries encoding the heavy (Fd fragment) or light (κ or λ) chains. The pGemT cloned PCR products were reamplified using two oligucleotide primer sets for introducing restriction sites for library cloning. A set of macaque κ oligonucleotide primers [21] or of human λ oligonucleotide primer (table 1) were used as forward (annealing to the 5' end of VH or VL) oligonucleotide primers. Only macaque-specific oligonucleotide primers were used as reverse oligonucleotide primers ([21] and table 1). The secondary PCRs were carried with separated forward oligonucleotide primers in order to maintain diversity. Each PCR was performed in 100 μl using 100 ng purified PCR reaction of the pGemT cloned cDNA, 4 U Red Taq (Sigma, Hamburg, Germany), 200 μM dNTPs each and 200 nM of each oligonucleotide primer for 20 cycles (30 s 94°C, 30 s 57°C, 30 s 72°C), followed by 10 min 72°C. The PCR products were separated by a 1.5% (w/v) agarose gel, then cut out and purified using Nucleospin Extract II Kit (Macherey-Nagel, Düren, Germany) according to the manufacturer's instructions.

Construction of the library was performed in two steps. First, the VLκ or VLλ PCR products were cloned to pHAL14 [21,28,29] and second, the VH PCR products were cloned to pHAL14 containing the VLκ or VLλ repertoire. The pHAL14 (5 μg), as well 2 μg VL (VLκ or VLλ), were digested using 50 U MluI and 50 U NotI (NEB, Frankfurt, Germany) in a 100 μl reaction volume (2 h at 37°C). The digest was inactivated by heating at 65°C for 10 min. Afterwards, 0.5 U calf intestinal phosphatase (MBI Fermentas) was added to the digested pHAL14 and incubated for an additional 30 min. This step was repeated once. The vector was purified using the Nucleospin Extract II Kit and 270 ng VL were cloned into 1 μg of the dephosporylated pHAL14 by incubating overnight (16°C) with 1 U ligase (Promega, Mannheim, Germany). The ligation reactions were precipitated with ethanol plus sodium acetate, and the pellet washed two times with 70% ethanol. These reactions were electroporated (1.7 kV) in 25 μl XL1-Blue MRF' (Stratagene, Amsterdam, Netherlands). The transformed bacteria were plated onto SOB agar plates (25 cm petri dishes) supplemented with 100 μg/mL ampicillin and 100 mM glucose. These colonies were harvested by suspending in 40 mL SOB media. Plasmids from the VLκ or VLλ library were isolated using the Nucleobond Plasmid Midi Kit (Macherey-Nagel) according to the manufacturer's instructions. Five micrograms of each VL chain library, as well 1.5 μg of the VH fragments, were digested using 50 U NcoI and 100 U HindIII (NEB) in a 100 μl reaction volume (overnight at 37°C). The following steps were performed as described for VL with the following modification: 250 ng of the digested and purified VH repertoire was used for ligation. In total 2 transformations were performed. The harvested bacteria, containing the final antibody gene libraries, were aliquoted and stored at -80°C.

For library packaging, 500 mL 2xTY medium [30] containing 100 μg/mL ampicillin and 100 mM glucose were inoculated with a 1 mL aliquot of the antibody gene library. Bacteria were grown (37°C, 250 rpm rotary shaker incubator) to an O.D._600 _of 0.4 – 0.5. Twenty-five millilitres of bacteria (~1,25 × 10^10^) were infected with 2,5 × 10^11 ^Hyperphage [31-33], incubated at 37°C for 30 min without shaking, followed by 30 min at 250 rpm. The infected cells were harvested by centrifugation (10 min, 3,220 × g). The pellet was resuspended in 200 mL 2xTY containing 100 μg/mL ampicillin and 50 μg/mL kanamycin. The phages were produced at 30°C and 250 rpm for 16 h. Cells were pelleted by centrifugation for 10 min (10,000 × g). The supernatant containing the phages was precipitated with 1/5 volume of 20% (w/v) polyethylene glycol (PEG)/2.5 M NaCl solution (1 h on ice) with gentle shaking and then pelleted by centrifugation for 1 h at 10,000 × g (4°C). The precipitated phage were resuspended in 10 mL phage dilution buffer (10 mM TrisHCl pH7.5, 20 mM NaCl, 2 mM EDTA) and filtered through a 0.45 μM filter. Phage precipitation was repeated once more. The phage pellet was diluted in 1 mL phage dilution buffer and cell debris was pelleted by additional centrifugation (5 min. at 15,400 × g, 20°C). The supernatant containing the phage were stored at 4°C. Each library packaging was controlled by tittering, and subsequently tested by immunoblot according to [28,34].

Screening of the library was performed as described elsewhere [35,36], except that 10, 15, and 30 washes were performed for each successive round of panning. Ricin was utilized as the antigen and PBS-Tween 20 0.1% as the washing buffer.

### scFv production, ELISA testing

Phagemid DNA isolated after the panning process was used to transform the non-suppressor *E. coli *strain HB2151 such that it expressed the soluble scFv fragment. Single colonies of randomly chosen transformants were used to inoculate 5 ml of SB (Super Broth) medium supplemented with carbenicillin (50 μg.ml^-1^) and 1% glucose. In parallel (see below), the clones were sequenced so that redundant clones were not re-analyzed. For expression, cultures were incubated overnight (30°C) with vigorous shaking (250 rpm). Five hundred milliliters of SB medium supplemented with carbenicillin and 0.1% glucose were then inoculated with 500 μl of each culture. The cultures were grown at 30°C until the 600 nm optical density (OD) reached a value of one. In order to induce gene expression, IPTG (1 mM) was added and the cultures incubated overnight (22°C). The cells were harvested by centrifugation at 2500 × g for 15 min, 4°C. The scFvs were extracted with polymyxin B sulphate [37] and purified using a nickel column (Ni-NTA spin column, Qiagen, Valencia, CA) according to the manufacturer's instructions.

For ELISAs, RCA-A or ricin (Sigma, 10 μg/ml PBS) was coated onto a 96 well microtitre plate (Maxisorp, Nunc, Danemark). When sera were tested, the negative control was pre-immune serum and secondary antibody reporter utilized polyclonal anti-macaque IgG, Fc specific tagged with HRP (Nordimmune, Tilburg, The Netherlands). The antibody titre was measured as the reciprocal of the highest dilution of immune serum giving a signal three times stronger than the negative control, at the same dilution. When scFv were tested, they were detected by an anti-his tag antibody (Qiagen, Courtaboeuf, France).

### Ricin neutralization in cell-based assays

Soluble scFvs, eluted after the library panning and expressed individually, were tested for their neutralization capacity using a cell-based viability assay. In this assay, cells are put in contact with ricin, in such conditions that all cells die. The scFv to be tested is added, at different concentrations, to ricin and may inhibit its toxic activity. At the end of the assay, cell viability is assessed and plotted against scFv concentration. This assay replicates the two main steps of ricin inhibition (internalization of the scFv bound to ricin and inhibition of its toxic activity). More precisely here, J774A.1 cells (ATCC-LGC, Molsheim, France) were plated at a density of 14,000 cells/well (200 μl/well), cultured (37°C with 5% CO_2_) for 24 hours in DMEM supplemented with 10% SVF. Ricin, at a concentration of 15 ng/ml (corresponding to 10 LD_50_, data not shown), was pre-incubated with scFvs, or with control serum for 1 h (37°C) and then added to the cells. After a 24 h incubation (37°C, 5% CO2), cell viability was determined by using the Cytotox 96 Non-radioactive Cytotoxicity Assay (Promega, Madison, Wi), as suggested by the manufacturer. Each assay was corrected for 100% cell viability (control wells with no toxin and no scFv) and 0% viability (control wells with ricin and no scFv). The scFv concentration corresponding to 50% viability is defined as the IC_50 _(inhibitory concentration 50%). These assays were performed three times in triplicate. All experiments were conducted on J774A.1 cells sub-cultured less than 15 times after receiving them from the vendor.

### ScFv stability

The scFv stability was estimated by determining the percentage of scFv active after a 24 h incubation at 37°C in DMEM medium. ScFvs (50 μg/ml in PBS) were incubated in triplicate and then tested in an ELISA, utilizing a freshly thawed aliquot for control.

### Ricin neutralization in a cell-free translation assay

In this assay, a cell-free translation system is put in contact with ricin, resulting in translation inhibition. The scFv is added, at different concentrations, to ricin and may inhibit its toxic activity. At the end of the assay, translation activity is plotted against scFv concentration. This assay replicates the intra-cellular step of ricin inhibition. More precisely here, the neutralizing activity of the best scFv was determined using a microtitre cell-free translation assay that detects luciferase translation from luciferase m-RNA [38]. By using phosphate buffered saline (PBS), antibodies were diluted in a 96 well microtitre plate and a constant amount of ricin (4 nM final concentration) was added to the antibody dilutions. Dilutions of ricin alone were included in each assay for generation of a standard. In addition, anti-ricin IgGs, purified from serum of mice that had been hyperimmunized with the dgRCA-A to result in antibodies with a high neutralization activity of ricin, were utilized as a benchmark of neutralization. The plate was placed on a microplate shaker for 15 min (25°C), and then 5 μl transferred to a v-shaped microtitre plate. The rabbit reticulocyte lysate, RNasin, amino acids complete, and luciferase m-RNA (Promega, Madison, WI) were mixed together and 25 μl added to each well of the v-shaped plate. Plates were incubated (37°C) for 90 min. Five microliters of the translation lysate were transferred to a black microtitre plate (Sigma Chemicals, St. Louis, MO). After the addition of 45 μl reaction buffer (Luciferase assay reagent, Promega, Inc.), luminescence was measured as counts per second (CPS) on a Victor multiplate reader (PerkinElmer Wallac, Boston MA). Data was expressed as the percent of control [% control = (CPS treated/CPS control) × 100].

### Affinity measurements

Affinities were measured by surface plasmon resonance (SPR) with a BIAcore™ × (Biacore, Uppsala, Sweden) instrument. The scFv 43RCA was immobilized at a maximum of 100 RU on a CM5 chip (Biacore) via amine coupling, according to the manufacturer's instructions. A 30 μl/min flow rate was maintained during measurement. For each measurement, eight ricin dilutions were prepared in HBS-EP buffer (Biacore) (concentrations ranging from 1 to 250 nM) and were tested at various elution times from 600 sec to 14,500 sec. For the precise measurement of very high affinities, we were advised (C. Quetard, Biacore, France) to use long elution times with the highest tested ricin concentration. After each ricin dilution, the chip was regenerated with 1.5 M glycine buffer (Biacore), run 10 μl/min for 30 seconds. Constants were calculated [39], and verified with internal consistency tests [40] as formerly described.

### Nucleic acid analysis of ricin-specific scFv clones

In parallel to expression, transformed HB2151 bacteria were cultivated to isolate the phagemidic DNA (Nucleobond AX, Macherey-Nagel). Variable region sequences of the light and heavy chains were determined by Genome Express (Meylan, France) using the primers Mkmyc and MkpelB, respectively [41]. The sequences were analyzed on line, using the International ImMunoGeneTics information system ^® ^(IMGT) [42] and its nomenclature. In particular, these sequences were compared with those of the human germline immunoglobulin genes by using the IMGT/V-QUEST [43] and IMGT/JunctionAnalysis softwares [44]. Peptidic sequence comparisons utilized new IMGT tools [26], which classify and compare three separate items; hydropathy, volume, and chemical properties, for each amino acid. This three item analysis was summarized as follows: when no significant difference between two residues was recognised in the three items, residues were regarded as highly similar. When one difference was found, the residues were regarded as similar but then regarded as dissimilar in the case of two differences, and very dissimilar in the case of three differences.

In addition, the similarities between all analysed scFv sequences were analysed using a new on-line tool , which builds phylogenetic trees. Phylogenetics in the strict sense apply to immunoglobulins because, due to somatic hypermutations that are the support for affinity maturation, unmutated rearranged germline sequences are ancestors of those coding for high affinity antibodies. After library panning however, only high affinity, mutated antibody fragments are retained. Thus, in this study, we utilised this tool as a multiple alignment software that displays results in the form of phylogenetic trees or sub-trees, and applied it to the sequences coding scFvs selected from the library. In such trees, each node, where certain branches of a tree meet, indicates the consensus shared by the different sequences displayed at the extremity of corresponding branches. The length of each branch, between each node and each sequence, is in proportion with the differences between the corresponding consensus and sequence.

### Nucleotide sequences

The *Macaca fascicularis *43RCA-H and 43RCA-L sequences (VH and VL domain sequences, respectively) are accessible in the data banks under the accession numbers [EMBL: FJ178346 and EMBL: FJ178347].

## Abreviations

Ab: antibody; BSA: bovine serum albumin; BW: bioweapon; HACA: human anti-chimeric antibody; IC_50_: concentration inhibiting 50% of toxicity; OD: optical density; NHP: non human primate; RCA-A: (*Ricinus communis *agglutinin, chain A) A chain of ricin; scFv: single chain Fv; VL: variable region of light chain; VH: variable region of heavy chain.

## Authors' contributions

TP performed the majority of the bench work except for the cloning of the library in pHAL14 and the cell-free assays. He participated in the presentation of scFv diversity in the form of trees and in the manuscript redaction. MH and SD cloned the library in their phagemidic vector, pHAL14, starting with the sublibraries, and wrote the corresponding paragraphs. MH performed the cell-free assays and wrote the corresponding paragraphs. She verified the manuscript. MPL verified the sequence analysis realized with IMGT, the server she conceived and maintains, and participated in the manuscript redaction. PT conceived the project, participated in the bench work except for the cloning of the library in pHAL14 and the cell-free assays, in the presentation of scFv diversity in the form of trees. He wrote the manuscript except for the paragraphs regarding the work performed by MH and SD, or by MH. All authors read and approved the final manuscript.
